# Tunable Polariton Rabi Oscillation in Phase‐Changing Perovskite Microcavities

**DOI:** 10.1002/advs.202417596

**Published:** 2025-03-17

**Authors:** Hyeon‐Seo Choi, Minjee Ko, Taejin Lee, Jin‐Woo Jung, Young‐Jun Lee, Hyeonjong Jeong, Youngjae Kim, Dongha Kim, Jinhee Heo, Shinbuhm Lee, JaeDong Lee, Chang‐Hee Cho

**Affiliations:** ^1^ Department of Physics and Chemistry Daegu Gyeongbuk Institute of Science and Technology (DGIST) Daegu 42988 South Korea; ^2^ Materials Analysis and Evaluation Department Korea Institute of Materials Science Changwon 51508 South Korea

**Keywords:** ferroicity, microcavity, phase transition, rabi oscillation

## Abstract

Exciton‐polaritons are composite quasiparticles hybridized between excitons and photons, which are very promising to develop quantum information devices such as entangled photon pair sources and polariton qubit devices by utilizing the fascinating properties of strong nonlinearity, Bose‐Einstein condensation, and superfluidity. Organic–inorganic hybrid lead halide perovskites have attracted much interest in cavity quantum electrodynamics due to their excellent excitonic properties, including strong exciton binding energy and high oscillation strength. Here, tunable Rabi oscillation of exciton‐polaritons in the lead halide perovskite microcavity is demonstrated, which experiences a phase transition between orthorhombic, tetragonal, and cubic phases by varying the temperature. Over the phase transition, the Rabi frequency is probed by tracing the dispersion relation of the exciton‐polaritons using Fourier plane spectroscopy. Due to the emergence of ferroelectricity in the tetragonal phase of the perovskites, the Rabi splitting can be tuned by ≈20%, while the corresponding exciton oscillator strength is varied by ≈44%. These results provide insight into novel functionalities of polariton devices by utilizing ferroic semiconductors, which can facilitate the development of tunable quantum devices.

## Graphical abstract

This work demonstrated the tunable Rabi oscillation of exciton‐polaritons in MAPbBr_3_ perovskites due to the emergence of ferroelectricity during the crystallographic phase transition. Both the Rabi oscillation and exciton oscillator strength exhibit an unusual temperature dependence that is related to the emergence of ferroelectricity in the tetragonal phase, showing anti‐correlation behavior between the exciton oscillator strength and the dielectric polarization.

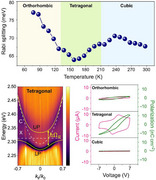

## 1 Introduction

Exciton‐polaritons are bosonic quasiparticles that arise from the quantum hybridization between the excitons in semiconducting materials and the confined photons in optical cavities, which can be achieved when the rate of energy exchange between the exciton and the cavity photon surpasses the energy dissipation in the system.^[^ ^1^, ^2^ ^]^ As a result, the exciton‐polaritons inherit a part‐light and part‐matter nature, with a small effective mass from their photonic components and strong nonlinearity from their excitonic components.^[^ ^1^ ^]^ These fascinating properties are critically important not only in macroscopic spontaneous coherence, including Bose–Einstein condensation ^[^ ^3^ ^]^ and superfluidity,^[^ ^4^, ^5^ ^]^ but also in practical applications, such as ultralow threshold lasers^[^ ^6^, ^7^, ^8^ ^]^ and quantum information devices utilizing strong nonlinearity of polaritons.^[^ ^9^, ^10^, ^11^ ^]^ Despite the maturity of fabrication technologies for microcavity devices based on conventional semiconductors of GaAs,^[^ ^6^ ^]^ the macroscopic coherence of exciton‐polaritons has been observed mostly at cryogenic temperatures, primarily due to the small exciton binding energies (≈10 meV). Consequently, alternative systems capable of room‐temperature operations need to be explored.

For room‐temperature polaritonic systems, wide‐gap inorganic semiconductors, such as GaN^[^ ^12^ ^]^ and ZnO,^[^ ^13^ ^]^ have been investigated as active media with exciton binding energies of a few tens of meV and needing complex quantum well structures to enhance the excitonic quality. Organic semiconductors have also been explored as alternatives due to the large binding energy (≈1 eV) of Frenkel excitons.^[^ ^14^ ^]^ However, weak exciton–exciton interactions, limited by the small Bohr radius of Frenkel excitons, can lead to a slight polariton nonlinearity and hinder many important polaritonic phenomena and applications. More recently, organic–inorganic lead halide perovskite semiconductors (MAPbX_3_; MA = CH_3_NH_3_^+^, X = Cl, Br, I) have emerged as promising candidates for room‐temperature polaritonic devices. These perovskite semiconductors show Wannier–Mott excitons possessing a high excitonic oscillator strength and large exciton binding energy with a large Bohr radius of a few nanometers,^[^ ^15^, ^16^, ^17^ ^]^ which can provide strong polariton nonlinearities even at room temperature. Furthermore, the lead halide perovskites provide rich tunability, such as bandgap engineering^[^ ^17^ ^]^ and the manipulation of dimensionality,^[^ ^18^, ^19^ ^]^ facilitating adaptation in realistic polariton devices.

In particular, the lead halide perovskites can undergo a crystallographic phase transition between orthorhombic, tetragonal, and cubic phases with a variation in the temperature.^[^ ^20^ ^]^ Importantly, ferroic properties can emerge due to a symmetry‐breaking distortion of lattices during the structural phase transition. This transition can induce a large variation and reversible switching of the physical properties, such as mechanical strain and electric polarization of crystal lattices.^[^ ^21^ ^]^ Moreover, ferroicity, such as ferroelectricity and ferroelasticity, has been observed in the lead halide perovskite semiconductors, showing spontaneous lattice polarization that can be switchable under external stimuli.^[^ ^22^, ^23^, ^24^ ^]^ This ferroicity can be utilized to produce tunable exciton‐polariton systems that can control the properties of exciton‐polaritons, including Rabi oscillation, oscillator strength, polarization, and coherence. Thus, these systems can facilitate advanced quantum polaritonic devices toward quantum simulators^[^ ^25^ ^]^ and quantum communications.^[^ ^9^, ^26^ ^]^

In this study, we investigate the tunable Rabi oscillation of exciton‐polaritons in hybrid lead halide perovskites of MAPbBr_3_ due to the emergence of ferroicity during the phase transition. The phase‐dependent Rabi oscillation is probed by tracing the dispersion relation of the exciton‐polaritons as a function of the temperature using Fourier plane spectroscopy, resulting in the corresponding phase‐dependent exciton oscillator strength. Both the Rabi oscillation and the exciton oscillator strength exhibit an unusual temperature dependence during the phase transition, exhibiting a significant decrease in the tetragonal phase. We also characterize the dielectric polarization using *I–V* and *P–V* hysteresis loop measurements, showing anti‐correlation behavior between the exciton oscillator strength and the dielectric polarization due to the ferroelectricity. Furthermore, the theoretical calculations suggest that the phase‐dependent Rabi oscillation and exciton oscillator strength originate from the ferroelectric domains in the tetragonal phase.

## 2 Results and Discussion

### 2.1 Optical Microcavities with Phase‐Changing MAPbBr_3_ Perovskites

**Figure 1:**
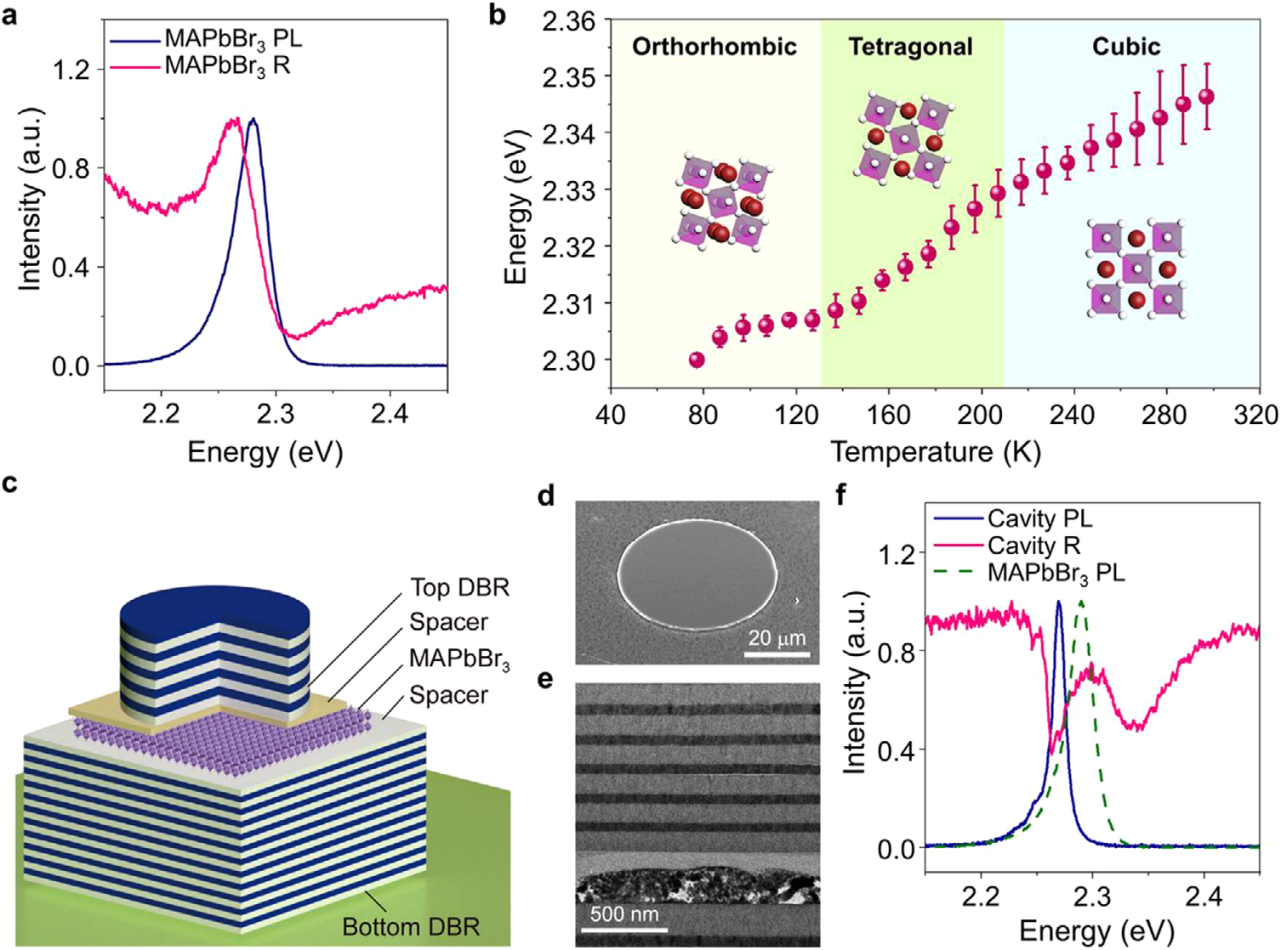
Schematic of the MAPbBr_3_ microcavity and characterization. a) Reflectance (pink) and photoluminescence (navy) of the MAPbBr_3_ thin film spin‐coated on a SiO_2_/Si substrate measured at 77 K. b) Temperature‐dependent exciton energy of the MAPbBr_3_ thin films. c) Schematic representation of the MAPbBr_3_ microcavity with the planar bottom DBR consisting of 11.5 pairs of ZnS/MgF_2_ and the micropatterned top DBR consisting of 5.5 pairs. d) Scanning electron microscopy showing the top DBR of the fabricated microcavity. e) Cross‐sectional transmission electron microscopy image showing the dry‐transferred top DBR upon the spacer layer above the MAPbBr_3_ thin film. f) Photoluminescence (navy solid line) and reflectance (pink solid line) spectra of the MAPbBr_3_ microcavity measured at 77 K. For comparison, the photoluminescence spectrum (olive dashed line) of the MAPbBr_3_ thin film on the SiO_2_/Si substrate is also shown.

The perovskite thin film of MAPbBr_3_ was used as an active material, possessing a strong excitonic resonance. As shown in **Figure** **1**a, the photoluminescence spectrum exhibits the excitonic emission peak at ≈2.289 eV at 77 K, while the reflectance shows the typical resonance feature at the exciton energy. The exciton energy was determined from the reflectance spectrum. The difference between the excitonic resonance in reflectance and the photoluminescence peak energies arises due to the phonon‐involved emission processes.^[^ ^27^ ^]^ The exciton binding energy was estimated to be ≈41 meV by fitting the absorption spectra using the Elliott model (Figure S1, Supporting Information).^[^ ^28^ ^]^ The exciton binding energies of Br and Cl halide perovskites have been reported to be larger than room temperature thermal energy (26 meV); thus, the exciton state can be thermally stable at room temperature.^[^ ^16^ ^]^ Interestingly, MAPbBr_3_ experiences a crystallographic phase transition from orthorhombic to tetragonal to cubic phases with increasing temperature.^[^ ^20^ ^]^ Figure 1b displays the temperature‐dependent exciton energy during the phase transitions; an overall blueshift is observed except for the small redshifts around the phase transition temperatures of ≈130 K from the orthorhombic to the tetragonal phase (Figure S2, Supporting Information). The crystallographic phase transition of MAPbBr_3_ is also confirmed by X‐ray diffraction analysis (Supporting Information S1 and Figure S3, Supporting Information). The cubic phase primally shows diffraction peaks of {100} crystal planes, whereas both the orthorhombic and tetragonal phases show diffraction peaks corresponding to the {110} and {002} planes.^[^ ^29^ ^]^ To investigate the exciton‐polariton properties for the different crystalline phases, MAPbBr_3_ was integrated with planar microcavities, which could support Fabry–Pérot cavity modes (Figure 1c). Our microcavity devices consisted of a MAPbBr_3_ film, PMMA (poly(methyl methacrylate)) as a top spacer layer, MgF_2_ as a bottom spacer layer, and top (5.5 pairs) and bottom (11.5 pairs) ZnS/MgF_2_ distributed Bragg reflectors (DBRs) with alternating layers of 110 nm‐thick MgF_2_ and 59 nm‐thick ZnS thin films. The MAPbBr_3_ thin film with a thickness of ≈210 nm and an average grain size of ≈160 nm was deposited by the nanocrystal pinning method on the prepared bottom DBR with an 186 nm‐thick MgF_2_ spacer.^[^ ^30^ ^]^ Another thin layer of 89 nm‐thick PMMA was then spin‐coated on the MAPbBr_3_ thin film as the spacer layer; this layer was used to determine the resonance frequency of cavity photon modes. Finally, the micropatterned top DBRs with a diameter of 50 µm were dry‐transferred onto the spacer layer to form the 3λ/2 microcavity devices, as shown in Figures 1d,e. The resonance modes of microcavity devices were calculated using the finite‐difference time‐domain (FDTD) method. The cavity confines three anti‐nodes within the DBR mirrors with a Q‐factor of 842 and a spatial overlap between the gain medium and photons of 0.24 (Figure S4, Supporting Information). Figure 1f shows the photoluminescence and reflectance spectra measured at 77 K to investigate the optical properties of the MAPbBr_3_ microcavity. The reflectance spectrum for normal incidence clearly exhibits upper and lower polariton (UP and LP) states at 2.339 and 2.268 eV, respectively, while showing the emission from the LP states in photoluminescence, indicating the strong coupling between MAPbBr_3_ excitons and cavity photons.

### 2.2 Phase‐Dependent Exciton‐Polariton Dispersion

**Figure 2:**
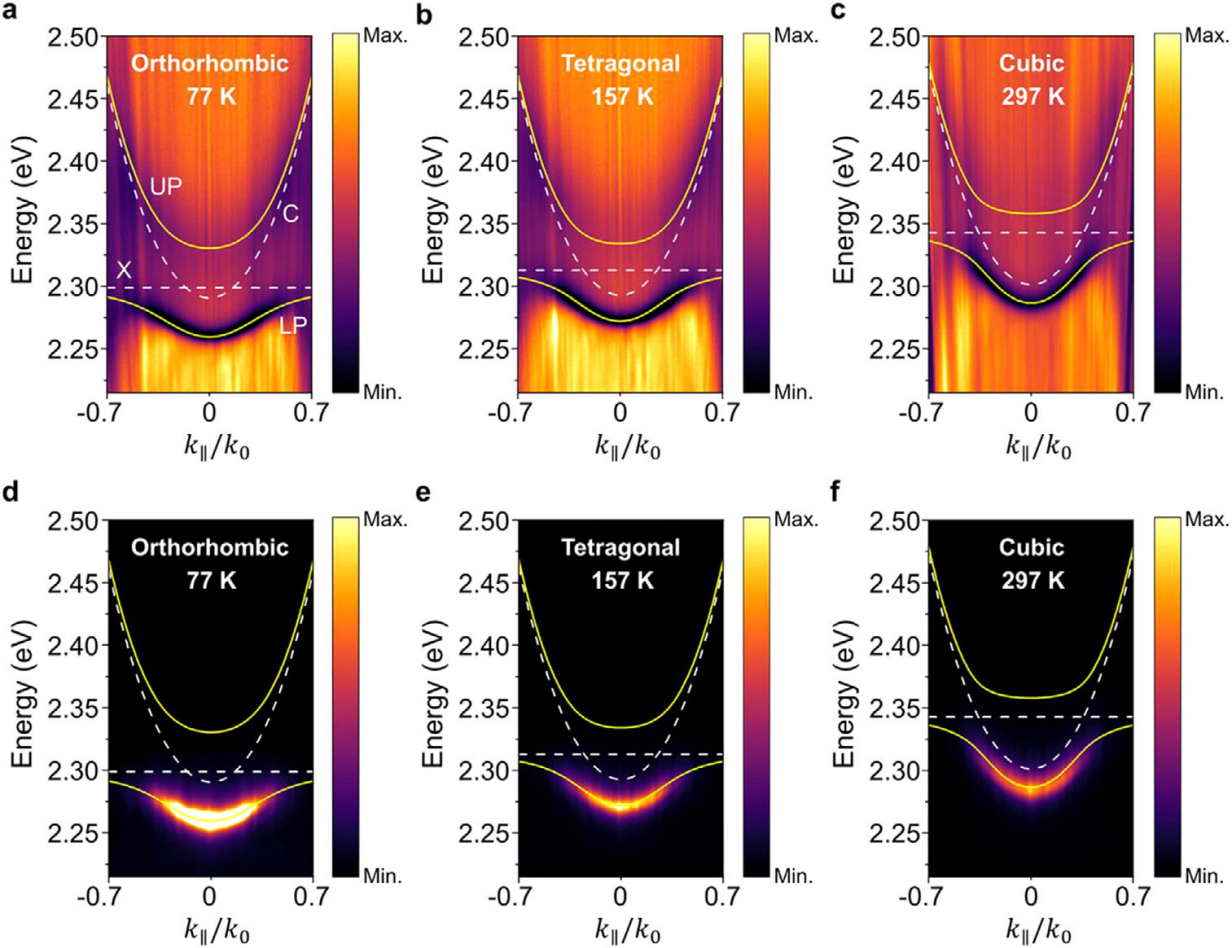
Phase‐dependent angle‐resolved reflectance and photoluminescence. a–c) Angle‐resolved reflectance spectra of the MAPbBr_3_ microcavity at orthorhombic (a), tetragonal (b), and cubic phases (c). Dashed white lines show the dispersion of uncoupled exciton (X) and cavity photon modes (C). Solid yellow lines display the dispersion of the UP and LP branches fitted using the coupled oscillator model. d–f) Angle‐resolved photoluminescence spectra of the MAPbBr_3_ microcavity at orthorhombic (d), tetragonal (e), and cubic phases (f). Fitted lines from the coupled oscillator model are overlaid with the photoluminescence spectra to compare with their corresponding reflectance spectra.

To study the evolution of the exciton‐polariton states in the MAPbBr_3_ microcavities of which MAPbBr_3_ experiences the crystallographic phase transition, the polariton dispersions are characterized by varying the temperature from 77 to 297 K. For both the reflectance and photoluminescence, the angle‐resolved signals are imaged at Fourier planes using a lab‐built microscope system. **Figure** **2**a–c displays the angle‐resolved reflectance spectra, showing the polariton dispersions for the orthorhombic (T = 77 K), tetragonal (T = 157 K), and cubic (T = 297 K) phases. The dispersion of the cavity photon (C; white dashed curves in Figure 2) can be described by $E_{ph}\;\left(\theta \right)=\left(E_{X}+\delta \right)/\sqrt{1-\sin^{2}\left(\theta \right)/n_{eff}^{2}}\;$, where *δ* is the detuning of the microcavity defined by *δ*  = *E_ph_* (0) − *E_X_*; *E_ph_* (0) is the cavity photon energy at normal incidence; *E_X_* is the exciton energy (X; white dashed lines in Figure 2); and *n_eff_* is the effective refractive index of the cavity. The exciton resonance energies were extracted from the temperature‐dependent reflectance spectra of the MAPbBr_3_ films (Supporting Information S2 and Figure S2, Supporting Information). The angle *θ* and the in‐plane wave vector *k*_∥_ have the relationship of *k*_∥_ = *k*_0_ sin (*θ*), where *k*_0_ is a wave vector in a vacuum. To explicitly assign the cavity photon dispersion, *n_eff_* was estimated by the best fitting of the uncoupled cavity photon mode lying higher than the exciton energy in the measured angle‐resolved reflectance spectra (Figures S5 and S6, Supporting Information). For all angle‐resolved reflectance spectra, two dispersion branches of UP and LP states clearly appear with anti‐crossing features between the cavity photon and the MAPbBr_3_ exciton dispersions, which is a typical signature of strong coupling. These exciton‐polariton dispersions can be described by the coupled oscillator model as follows:^[^ ^31^ ^]^

(1) $$\left(\begin{array}{cc} E_{X}+i\hbar \gamma_{X} & g\\ g & E_{ph}\left(\theta \right)+i\hbar \gamma_{ph} \end{array}\right)\left(\begin{array}{c} \alpha \\ \beta \end{array}\right)=E\left(\begin{array}{c} \alpha \\ \beta \end{array}\right)$$

where γ_*X*_ and γ_*ph*_ are the decay rates of the exciton and cavity photon, respectively; *g* is the coupling strength between the exciton and photon; *E* is the eigenvalue of the coupled system; and *α* and *β* represent the eigenvectors (|*α*|^2^ + |*β*|^2^ = 1). Using the coupled oscillator model (Supporting Information S2), the fitted polariton dispersions are exactly overlaid with the measured reflectance spectra (shown as yellow solid curves in Figure 2). Figure 2d−f shows the angle‐resolved photoluminescence spectra; dominant photoluminescence is observed from the LP branch due to a much faster scattering time from the UP to the LP states compared to the polariton lifetime. Notably, the polariton dispersion curves obtained from the reflectance spectra are well consistent with the angular distributions of photoluminescence. As the temperature increases, the exciton resonance prominently shifts to higher energies along with a small change in the cavity photon mode, resulting in the variation in detuning of the MAPbBr_3_ microcavities. For the orthorhombic (T = 77 K), tetragonal (T = 157 K), and cubic (T = 297 K) phases, the detuning is estimated to be −8, −20, and −42 meV, respectively (Figure 2). This result implies that the phase‐dependent evolution of exciton‐polaritons can be traced by changing the temperature of the MAPbBr_3_ microcavities.

**Figure 3:**
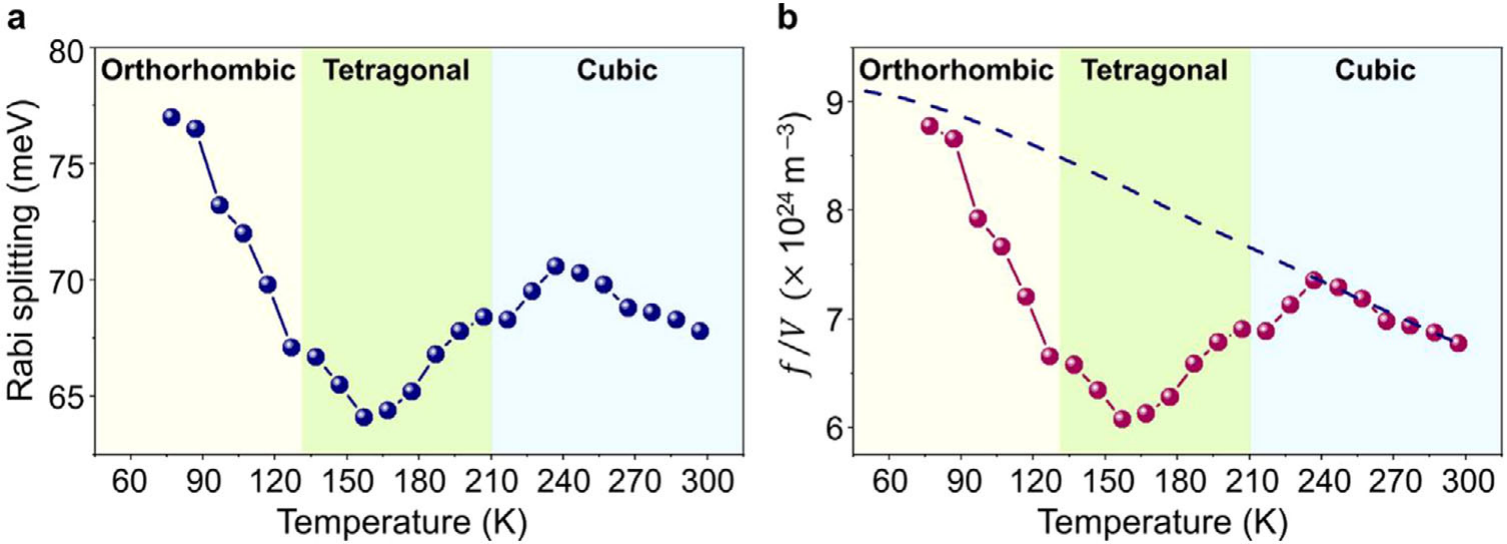
Tunable Rabi splitting energies of exciton‐polaritons in the MAPbBr_3_ microcavity. a) Rabi splitting energies of exciton‐polaritons as a function of temperature. b) Exciton oscillator strength extracted from the temperature‐dependent Rabi splitting energies of (a). The blue dashed line indicates the exciton oscillator strength for a typical semiconductor as a function of temperature calculated by the Debye‐Waller factor.

Importantly, the intrinsic properties of exciton‐polaritons can be modified by the crystallographic phase transition of MAPbBr_3_. For example, a phase‐dependent property, such as ferroicity, can emerge in a specific phase of MAPbBr_3_; this will be discussed later. This would enable the tuning of the exciton oscillator strength and, thus, the Rabi oscillation energies by the phase transition since the exciton oscillator strength can be significantly modified with the emergence of ferroelectricity. The exciton oscillator strength is related to the Rabi frequency through $f/V=\left(\varepsilon_{\infty }\varepsilon_{0}m_{0}\Omega_{R}^{2}\right)/\left(4\pi e^{2}\Gamma_{\mathrm{over}\mathrm{lap}}\right)\;$, where *f*/*V* is the oscillator strength per unit volume; *Ω*_*R*_ is the Rabi frequency; and *Γ*_*overlap*_ is the spatial overlap factor. To quantify the Rabi oscillation in the MAPbBr_3_ microcavity during the phase transition, we estimated the Rabi splitting energy (ℏ*Ω*_*R*_) as a function of the temperature from the measured polariton dispersions, as shown in **Figure** **3**a (Supporting Information S3). Interestingly, we observe a significant change in the Rabi splitting of exciton‐polaritons during the phase transition. First, for the orthorhombic phase corresponding to the measured temperature range from 77 to 130 K, the Rabi splitting energy is continuously decreased from 77 to 67 meV. Second, in the tetragonal phase ranging from 130 to 210 K, the Rabi splitting value becomes minimized with a value of 64 meV at a temperature of 157 K and then increases up to the transition temperature (≈210 K) to the cubic phase. Third, within the cubic phase corresponding to the temperature range above 210 K, the Rabi splitting value reaches a maximum of 237 K and then shows a monotonic decrease with increasing temperature. The significant variation in the Rabi splitting energy with unusual temperature dependence indicates that the intrinsic exciton property can be modified with the phase change of MAPbBr_3_. Using the measured Rabi splitting energies, we estimated the exciton oscillator strength (*f*/*V*) as a function of the temperature, as shown in Figure 3b. Considering the temperature (phase)‐dependent refractive index of MAPbBr_3_, the spatial overlap factor (*Γ*_*overlap*_) was calculated using FDTD simulations. The estimated exciton oscillator strength also shows a strong phase dependence, which is nearly the same as that of the Rabi splitting energy. The dashed line in Figure 3b indicates the temperature dependence of the exciton oscillator strength for typical semiconductors, showing a monotonic decrease due to exciton‐phonon scattering with increasing temperature (Supporting Information S4). In the phase‐changing MAPbBr_3_ microcavity, the Rabi splitting energy is tuned by ≈20%, while the corresponding exciton oscillator strength varies by ≈44% during the transition between the tetragonal and orthorhombic phases. Therefore, the unusual phase dependence of Rabi oscillation and exciton oscillator strength strongly suggests the emergence of ferroicity in MAPbBr_3_ during the crystallographic phase transition.

### 2.3 Ferroicity in Phase‐Changing MAPbBr_3_

**Figure 4:**
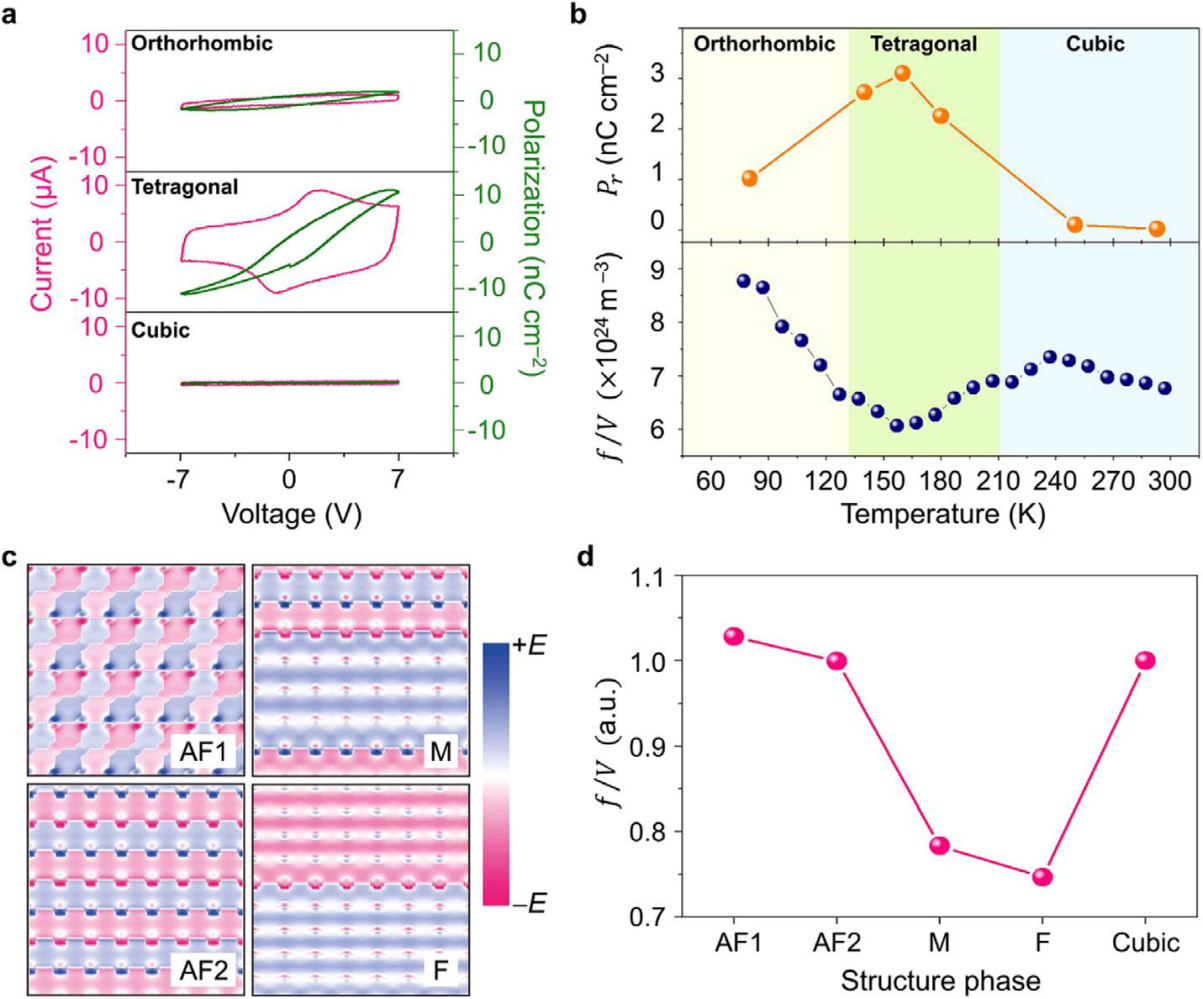
Phase‐dependent ferroelectricity of MAPbBr_3_ and theoretical model calculations of oscillator strengths. a) Current‐voltage loops (pink curves) and the corresponding polarization–voltage loops (olive curves) for orthorhombic (top panel), tetragonal (middle panel), and cubic phases (bottom panel). b) Remanent polarizations (orange spheres) calculated by half of the sum of the positive and negative polarizations at zero voltage as a function of temperature $(\;P_{r}=\left(|P_{r}^{+}\left|+\right|P_{r}^{-}|\right)/2)\;$(top panel). The bottom panel shows the exciton oscillator strength. c) Four types of polarization domains are used for theoretical modeling to investigate the effect of ferroelectricity on the exciton oscillator strength. Red (blue) indicates positive (negative) electric field strengths with *E* = 0.002 atomic units. AF1, AF2, M, and F denote an anti‐ferroelectric phase 1, anti‐ferroelectric phase 2, mixed‐phase, and ferroelectric phase, respectively. d) Calculated exciton oscillator strength for the anti‐ferroelectric, ferroelectric, mixed, and cubic phases normalized to that of the cubic structure.

To investigate the ferroelectric properties in MAPbBr_3_ depending on the phase transition, we carried out dielectric polarization measurements using fabricated capacitor‐type devices consisting of 100 µm‐thick MAPbBr_3_ crystals, Ag top electrodes, and Au bottom electrodes (Figure S7, Supporting Information). As shown in **Figure** **4**a, the *I–V* (pink solid line) and *P–V* (green solid line) hysteresis loops were measured for the orthorhombic, tetragonal, and cubic phases by varying the temperature along the sweep direction from zero (0 V) through positive (+7 V) to negative (−7 V) and then back to zero (0 V) voltages. The measured dielectric polarization also shows the explicit phase‐dependent behavior. For the orthorhombic phase (Figure 4a, top panel), a small elliptical loop is observed with a nonzero crossing point in the polarization, which is typical behavior for lossy capacitors. The small polarization value at zero voltage does not indicate a ferroelectric remanence in this lossy capacitor.^[^ ^21^ ^]^ However, for the tetragonal phase (Figure 4a, middle panel), the current peaks are observed in the *I–V* hysteresis loop, which can be attributed to the change in resistance by electric polarization switching. Accordingly, the remanent polarization (*P*_r_) appears in the *P–V* hysteresis loop. The observed current peak and remanent polarization indicate that the ferroelectricity emerges in the tetragonal phase of MAPbBr_3_. For the cubic phase (Figure 4a, bottom panel), the *I–V* and *P–V* curves show a negligible hysteresis, which can be observed in simple paraelectric materials. Figure 4b displays the measured remanent polarization $\left(\;P_{r}=\left(|P_{r}^{+}\left|+\right|P_{r}^{-}|\right)/2\right)$ in comparison with the exciton oscillator strength (*f*/*V*) as a function of temperature, where the remanent polarization is estimated by half of the sum of the positive and negative y‐intercept values $\left(|P_{r}^{+}\left|+\right|P_{r}^{-}|\right)$ of the *P–V* hysteresis curves. Clear anti‐correlation behavior is observed between the measured remanent polarization and exciton oscillator strength, showing maximized remanent polarization and minimized exciton oscillator strength in the tetragonal phase. This result strongly indicates that the exciton oscillator strength becomes significantly reduced by the formation of ferroelectric domains in the tetragonal phase of MAPbBr_3_.

To verify the observed ferroelectricity, we have performed second harmonic generation (SHG) measurements to provide evidence for the noncentrosymmetric nature of MAPbBr_3_ under a fs‐pulsed excitation at a wavelength of 806 nm. Unlike the centrosymmetric cubic phase, the SHG signals were clearly observed in both orthorhombic and tetragonal phases, indicating noncentrosymmetry, as shown in Figure S8a–c (Supporting Information). Additionally, the excitation‐polarization‐dependent measurements exhibited distinct patterns, showing the 2‐fold and 4‐fold rotational symmetry for orthorhombic and tetragonal phases, respectively (Figures S8d,e, Supporting Information).^[^ ^32^ ^]^ The existence of noncentrosymmetry implies that the polarity can be formed by the dipole moments in the lattice.^[^ ^33^ ^]^ The resulting electric polarization arises from the formation of polar domains with aligned dipole moments, known as spontaneous polarization. When the dipole moments are aligned in parallel, the ferroelectricity can be established, whereas an anti‐parallel alignment leads to the formation of anti‐ferroelectricity. In the tetragonal phase, the observed ferroelectricity with the remanent polarization and switching would result from the polar domains. In the case of the orthorhombic phase, it has been reported that MAPbBr_3_ crystals show anti‐ferroelectric behavior due to the anti‐parallel arrangement of polar MA^+^ cations, as revealed in the scanning tunneling microscope results at 4.5 K.^[^ ^34^ ^]^ The observed negligible remanent polarization can be attributed to the anti‐ferroelectricity in the orthorhombic phase. On the other hand, ferroelasticity, which stands for an elastic and reversible deformation under mechanical stress, has been suggested as a possible interpretation for the observed ferroic properties in orthorhombic and tetragonal MAPbBr_3_ crystals.^[^ ^35^ ^]^ To confirm the presence of ferroelasticity, the change in surface morphologies was imaged on bulk MAPbBr_3_ crystals with cleaved smooth surfaces during the phase transition.^[^ ^36^ ^]^ As shown in Figure S9 (Supporting Information), ferroelastic domains were observed as clear stripe patterns in both the orthorhombic and tetragonal phases that exhibited noncentrosymmetry. For the cubic phase, the MAPbBr_3_ crystals show a smooth surface morphology without any patterns. This implies that the observed ferroelastic domains would also possess a polar nature. In a polar ferroelastic domain, the net dipole moments can be established due to the displacement of atoms driven by the induced structural stress. These ferroelastic domains can be modulated indirectly by an external electric field through a structural deformation, possibly contributing to the weak electric response.^[^ ^37^ ^]^ Therefore, the observed ferroelastic domains could also contribute to the observed polarization behavior of MAPbBr_3_. As a result, our observation strongly suggests that both ferroelectricity and ferroelasticity coexist in the tetragonal phase of MAPbBr_3_.

To demonstrate quantified evidence for the underlying link between polar domains and exciton oscillator strengths, we employ first‐principles calculations to simply model crystals of an ideal semiconductor system and to simulate the ferroelectric MAPbBr_3_ (Supporting Information S5).^[^ ^38^ ^]^ The electrons of the model undergo external perturbative electric fields, *E_ext_* (*r*), corresponding to the polarization field of the individual domains of MAPbBr_3_, whose arrangements affect exciton formations and their oscillator strengths. Four types of polar domain configurations are displayed in Figure 4c. The case in which the *E_ext_* (*r*) is arranged to be maximally anti‐parallel with respect to the nearest neighbor cells, corresponds to the AF1 (anti‐ferroelectric 1) phase, whereas that of the AF2 (anti‐ferroelectric 2) phase belongs to the anti‐parallel arrangements only along the y‐direction. The M (mixed) phase is defined as a mixture of the AF2 and F (ferroelectric) phases. The F phase exhibits the largest homogeneous field in the ferroelectric domain. From the ground state calculations for the respective phases, we solve the Bethe‐Salpeter equation and compute the exciton oscillator strengths, as depicted in Figure 4d. First, the oscillator strength is found to decrease significantly as the configurational phase is changed from the anti‐ferroelectric phase to the mixed phase to the ferroelectric phase. Second, the cubic phase, where the polar domains are clearly eliminated, causes an exciton oscillator strength comparable to that of the anti‐ferroelectric phase. It is worth noting that one important variable related to the exciton oscillator strength is mainly attributed to the size of the polar electric domain area. Specifically, the widened homogeneity of the polar domains drastically suppresses a spatial overlap of electron and hole wavefunctions, and thus, the exciton oscillator strength becomes significantly reduced in the ferroelectric phase, while the increased inhomogeneity of the polar domains would gradually enhance the exciton oscillator strength in the mixed and anti‐ferroelectric phases (Figures S10 and S11, Supporting Information).

## 3 Conclusion

We demonstrated the tunable Rabi oscillation of exciton‐polaritons in the hybrid lead halide perovskites of MAPbBr_3_ due to the emergence of ferroicity during the phase transition. Both the Rabi oscillation and the exciton oscillator strength exhibited an unusual temperature dependence during the phase transition, showing a significant decrease and minimum values in the tetragonal phase due to the ferroelectricity. In the phase‐changing MAPbBr_3_ microcavity, the Rabi splitting energy was tuned by ≈20%, while the corresponding exciton oscillator strength was varied by ≈44%. The dielectric polarization measurements revealed a clear anti‐correlation between the exciton oscillator strength and the dielectric polarization. Furthermore, theoretical calculations suggested that the observed phase dependence of Rabi oscillation and exciton oscillator strength originated from the emergence of ferroelectric domains in the MAPbBr_3_ crystals. Our study provides novel degrees of freedom for polariton devices by utilizing ferroic semiconductors, and these polariton devices enable the development of tunable quantum polaritonics.

## 4 Experimental Section

### Sample Fabrication

MAPbBr_3_ nanograin films were synthesized using a well‐established anti‐solvent nanocrystal pinning method.^[^ ^30^ ^]^ To prepare the MAPbBr_3_ solution (1.0 m), MABr (Sigma–Aldrich) and PbBr_2_ (Sigma–Aldrich) were dissolved in anhydrous dimethyl sulfoxide (Sigma–Aldrich) within a glass vial. The solution was thoroughly stirred at 60 °C and 500 rpm for over 12 h and subsequently cooled down to room temperature. MAPbBr_3_ films were spin‐coated onto a substrate treated with O_2_ plasma. The substrate accelerated from 0 to 3600 rpm within 7 s, then held at 3600 rpm for 90 s. During the spin‐coating process, to reduce the grain size, an anti‐solvent (chloroform) of 100 µL was slowly dropped onto the film over 30 s before the spin‐coating process was completed. The sample was baked at 100 °C for 10 min. All procedures were performed under a nitrogen atmosphere in a glovebox. For the electrical and optical measurements, MAPbBr_3_ bulk crystals were also synthesized by inverse temperature crystallization.^[^ ^39^ ^]^ The solution was prepared using the same procedure, while the thoroughly solved solution was slowly heated from 30 to 100 °C over 12 h.

### Optical Characterization

Optical measurements of the MAPbBr_3_ nanocrystalline film and microcavities were conducted using a lab‐built microscope system integrated with objectives (Nikon) and also a liquid nitrogen‐cooled cryostat (Janis Research). The photoluminescence was measured using a continuous‐wave 457.9 nm argon laser (Coherent) focused by a 60× objective [NA (numerical aperture) = 0.7] with a beam spot size of ≈0.9 µm, while reflectance was measured by halogen fiber illuminator (Thorlabs) focused by the same objective lens to a beam spot size of ≈5 µm. The photoluminescence and reflectance signals of the perovskite films were collected through optical fiber coupled to a 0.5 m spectrometer (Acton) equipped with a cooled CCD (charged‐coupled device, Pixis 2K, Princeton Instruments) of 512 × 2048 pixels with a spectral resolution of 0.1 nm. The angle‐resolved spectroscopy was performed using the Fourier plane imaging setup. SHG measurements were measured using a Ti:sapphire laser (Coherent) at a repetition rate of 76 MHz and wavelength of 806 nm. The temperature‐dependent optical microscopy images were measured using white light sources focused by a 10× objective.

### Dielectric Hysteresis Measurements

The current‐voltage (*I–V*) and polarization‐voltage (*P–V*) measurements were performed on a Precision LC II ferroelectric tester (Radiant Technologies) under a vacuum condition of 10^−5^ torr to perform low‐temperature measurements to protect the samples from degradation. All temperature‐dependent measurements were performed using a vacuum probe station (NEXTRON) in a dark ambient condition. The *I–V* and *P–V* hysteresis loops were obtained in the voltage range of ±7 V at the frequency of 2 kHz.

## Conflict of Interest

The authors declare no conflict of interest.

## Author Contributions

H.‐S.C., M.K., T.L., and C.‐H.C. conceived and designed the project. H.‐S.C., M.K., and T.L. fabricated the samples. H.‐S.C., T.L., J.‐W.J., Y.‐J.L., H.J., and C.‐H.C. performed the optical measurements. H.‐S.C., J.‐W.J., and Y.‐J.L. performed the numerical simulations. Y.K. and J.D.L. developed the theory of exciton oscillator strength in the ferroelectric domains. H.‐S.C., M.K., D.K., J.H., S.L., and C.‐H.C. conducted electrical measurements and analyzed the data in the manuscript. H.‐S.C. and C.‐H.C. wrote the manuscript. All authors contributed to revising the manuscript and the supplementary materials.

## Supporting information



Supporting Information

## Data Availability

The data that support the findings of this study are available from the corresponding author upon reasonable request.

## References

[1] H. Deng , H. Haug , Y. Yamamoto , Rev. Mod. Phys. 2010, 82, 1489.

[2] C. Weisbuch , M. Nishioka , A. Ishikawa , Y. Arakawa , Phys. Rev. Lett. 1992, 69, 3314. 10046787 10.1103/PhysRevLett.69.3314

[3] J. Kasprzak , M. Richard , S. Kundermann , A. Baas , P. Jeambrun , J. M. J. Keeling , F. M. Marchetti , M. H. Szymańska , R. André , J. L. Staehli , V. Savona , P. B. Littlewood , B. Deveaud , L. S. Dang , Nature 2006, 443, 409. 17006506 10.1038/nature05131

[4] A. Amo , J. Lefrère , S. Pigeon , C. Adrados , C. Ciuti , I. Carusotto , R. Houdré , E. Giacobino , A. Bramati , Nat. Phys. 2009, 5, 805.

[5] G. Lerario , A. Fieramosca , F. Barachati , D. Ballarini , K. S. Daskalakis , L. Dominici , M. D. Giorgi , S. A. Maier , G. Gigli , S. Kéna‐Cohen , D. Sanvitto , Nat. Phys. 2017, 13, 837.

[6] H. Deng , G. Weihs , D. Snoke , J. Bloch , Y. Yamamoto , Proc. Natl. Acad. Sci. USA 2003, 100, 15318. 14673089 10.1073/pnas.2634328100 PMC307565

[7] C. Schneider , A. Rahimi‐Iman , N. Y. Kim , J. Fischer , I. G. Savenko , M. Amthor , M. Lermer , A. Wolf , L. Worschech , V. D. Kulakovskii , I. A. Shelykh , M. Kamp , S. Reitzenstein , A. Forchel , Y. Yamamoto , S. Höfling , Nature 2013, 497, 348. 23676752 10.1038/nature12036

[8] J.‐W. Kang , B. Song , W. Liu , S.‐J. Park , R. Agarwal , C.‐H. Cho , Sci. Adv. 2019, 5, eaau9338. 31016237 10.1126/sciadv.aau9338 PMC6474768

[9] D. Sanvitto , S. Kéna‐Cohen , Nat. Mater. 2016, 15, 1061. 27429208 10.1038/nmat4668

[10] C. Bennett , D. DiVincenzo , Nature 2000, 404, 247. 10749200 10.1038/35005001

[11] C. Ciuti , Phys. Rev. B 2004, 69, 245304.

[12] S. Christopoulos , G. B. Höger von Högersthal , A. J. D. Grundy , P. G. Lagoudakis , A. V. Kavokin , J. J. Baumberg , G. Christmann , R. Butté , E. Feltin , J.‐F. Carlin , N. Grandjean , Phys. Rev. Lett. 2007, 98, 126405. 17501142 10.1103/PhysRevLett.98.126405

[13] F. Li , L. Orosz , O. Kamoun , S. Bouchoule , C. Brimont , P. Disseix , T. Guillet , X. Lafosse , M. Leroux , J. Leymarie , G. Malpuech , M. Mexis , M. Mihailovic , G. Patriarche , F. Réveret , D. Solnyshkov , J. Zuniga‐Perez , Appl. Phys. Lett. 2013, 102, 191118. 10.1103/PhysRevLett.110.196406 23705728

[14] S. Kéna‐Cohen , S. R. Forrest , Nat. Photonics 2010, 4, 371.

[15] S. Zhang , Q. Shang , W. Du , J. Shi , Z. Wu , Y. Mi , J. Chen , F. Liu , Y. Li , M. Liu , Q. Zhang , X. Liu , Adv. Opt. Mater. 2018, 6, 1701032.

[16] R. Su , C. Diederichs , J. Wang , T. C. H. Liew , J. Zhao , S. Liu , W. Xu , Z. Chen , Q. Xiong , Nano Lett. 2017, 17, 3982. 28541055 10.1021/acs.nanolett.7b01956

[17] L. Protesescu , S. Yakunin , M. I. Bodnarchuk , F. Krieg , R. Caputo , C. H. Hendon , R. Xi Yang , A. Walsh , M. V. Kovalenko , Nano Lett. 2015, 15, 3692. 25633588 10.1021/nl5048779 PMC4462997

[18] Q. Zhang , R. Su , X. Liu , J. Xing , T. C. Sum , Q. Xiong , Adv. Funct. Mater. 2016, 26, 6238.

[19] C.‐H. Cho , C. O. Aspetti , J. Park , R. Agarwal , Nat. Photonics 2013, 7, 285. 23710256 10.1038/nphoton.2013.25 PMC3661302

[20] A. Poglitsch , D. Weber , J. Chem. Phys. 1987, 87, 6373.

[21] M. G. Cain , Characterisation of Ferroelectric Bulk Materials and Thin Films, 2nd ed., Springer, Dordrecht, Netherlands 2014.

[22] H. Röhm , T. Leonhard , A. D. Schulz , S. Wagner , M. J. Hoffmann , A. Colsmann , Adv. Mater. 2019, 31, 1806661. 10.1002/adma.201806661 30785225

[23] B. Huang , Z. Liu , C. Wu , Y. Zhang , J. Zhao , X. Wang , J. Li , Natl. Sci. Rev. 2021, 8, nwab094. 34691717 10.1093/nsr/nwab094 PMC8363338

[24] Z. R. Gao , X. F. Sun , Y. Y. Wu , Y. Z. Wu , H. L. Cai , X. S. Wu , J. Phys. Chem. Lett. 2019, 10, 2522. 31042032 10.1021/acs.jpclett.9b00776

[25] C. Schneider , K. Winkler , M. D. Fraser , M. Kamp , Y. Yamamoto , E. A. Ostrovskaya , S. Höfling , Rep. Prog. Phys. 2017, 80, 016503. 27841166 10.1088/0034-4885/80/1/016503

[26] S. S. Demirchyan , I. Yu. Chestnov , A. P. Alodjants , M. M. Glazov , A. V. Kavokin , Phys. Rev. Lett. 2014, 112, 196403. 24877953 10.1103/PhysRevLett.112.196403

[27] Y. Guo , O. Yaffe , T. D. Hull , J. S. Owen , D. R. Reichman , L. E. Brus , Nat. Commun. 2019, 10, 1175. 30862815 10.1038/s41467-019-09057-5 PMC6414684

[28] M. Saba , M. Cadelano , D. Marongiu , F. Chen , V. Sarritzu , N. Sestu , C. Figus , M. Aresti , R. Piras , A. G. Lehmann , C. Cannas , A. Musinu , F. Quochi , A. Mura , G. Bongiovanni , Nat. Commun. 2014, 5, 5049. 25266869 10.1038/ncomms6049

[29] S. Luo , W. A. Daoud , Materials 2016, 9, 123. 28773249 10.3390/ma9030123 PMC5456724

[30] H. Cho , S.‐H. Jeong , M.‐H. Park , Y.‐H. Kim , C. Wolf , C.‐L. Lee , J. H. Heo , A. Sadhanala , N. Myoung , S. Yoo , S. H. Im , R. H. Friend , T.‐W. Lee , Science 2015, 350, 1222. 26785482 10.1126/science.aad1818

[31] S. Haroche , D. Kleppner , Phys. Today 1989, 42, 24.

[32] K. Frohna , T. Deshpande , J. Harter , W. Peng , B. A. Barker , J. B. Neaton , S. G. Louie , O. M. Bakr , D. Hsie , M. Bernardi , Nat. Commun. 2018, 9, 1829. 29739939 10.1038/s41467-018-04212-w PMC5940805

[33] J. M. Frost , K. T. Butler , F. Brivio , C. H. Hendon , M. van Schilfgaarde , A. Walsh , Nano Lett. 2014, 14, 2584. 24684284 10.1021/nl500390f PMC4022647

[34] R. Ohmann , L. K. Ono , H.‐S. Kim , H. Lin , M. V. Lee , Y. Li , N.‐G. Park , Y. Qi , J. Am. Chem. Soc. 2015, 137, 16049. 26639900 10.1021/jacs.5b08227

[35] M. Bari , A. A. Bokov , Z. G. Ye , J. Mater. Chem. C 2021, 9, 3096.

[36] B. Zhang , S. Sun , Y. Jia , J. Dai , D. T. N. Rathnayake , X. Huang , J. Casasent , G. Adhikari , T. A. Billy , Y. Lu , X. C. Zeng , Y. Guo , Adv. Mater. 2023, 35, 2208336. 10.1002/adma.202208336 36493380

[37] D. Kim , J. S. Yun , P. Sharma , D. S. Lee , J. Kim , A. M. Soufiani , S. Huang , M. A. Green , A. W. Y. Ho‐Baillie , J. Seidel , Nat. Commun. 2019, 10, 444. 30683878 10.1038/s41467-019-08364-1 PMC6347646

[38] J. P. Perdew , Y. Wang , Phys. Rev. B 1992, 45, 13244. 10.1103/physrevb.45.13244 10001404

[39] M. I. Saidaminov , A. L. Abdelhady , B. Murali , E. Alarousu , V. M. Burlakov , W. Peng , I. Dursun , L. Wang , Y. He , G. Maculan , A. Goriely , T. Wu , O. F. Mohammed , O. M. Bakr , Nat. Commun. 2015, 6, 7586. 26145157 10.1038/ncomms8586 PMC4544059

