# Supplementary material for: Tunable Polariton Rabi Oscillation in Phase‐Changing Perovskite Microcavities
Source: Adv Sci (Weinh). 2025 Mar 17;12(20):2417596. doi: 10.1002/advs.202417596 (PMC12120701; doi:10.1002/advs.202417596)
Supplement: Supplementary file 1 — Supporting Information [file ADVS-12-2417596-s001.docx]

Supporting Information

Tunable Polariton Rabi Oscillation in Phase-Changing Perovskite Microcavities

Hyeon-Seo Choi, Minjee Ko, Taejin Lee, Jin-Woo Jung, Young-Jun Lee, Hyeonjong Jeong, Youngjae Kim, Dongha Kim, Jinhee Heo, Shinbuhm Lee, JaeDong Lee, Chang-Hee Cho*

**Supplementary Text**

**S1. Analysis of phase transition for MAPbBr_3_ films**

Figure S3 displays the temperature-dependent X-ray diffraction spectra for MAPbBr_3_ single crystalline microplates measured from 296 K to 81 K. In this temperature range, the MAPbBr_3_ crystal exhibits three distinct crystallographic structures of cubic, tetragonal, and orthorhombic phases.^[1]^ In the cubic phase, the {100} crystal planes predominantly exhibit diffraction peaks with high intensity, indicating the high symmetry of the crystal. However, in the tetragonal and orthorhombic phases, additional diffraction peaks corresponding to the {002} and {110} crystal planes emerge due to reduced symmetry resulting from changes in one or two lattice constants. In particular, the orthorhombic phases display larger differences between {002} and {110} diffraction angles.^[2]^ As shown in Figure S3, for the cubic phase (T > 210 K), the four prominent diffraction peaks corresponding to (100), (200), (300), and (400) planes are observed at around 15.0°, 30.2°, 46.0°, and 62.8°, respectively. However, for the tetragonal (210 K > T > 130 K) and orthorhombic (T < 130 K) phases, these main diffraction peaks split into two peaks of {002} and {110} planes. For the orthorhombic phase, the diffraction peaks of {002} and {110} planes still exist, but the gap between the two peaks is relatively wider compared to the tetragonal phase.

**S2. Dispersion of exciton-polaritons**

First, to determine the exciton energy and linewidth, we performed the best fitting using a least-squares method for the Lorentzian shape of reflectance spectra on MAPbBr_3_ films, as shown in Figure S2. The dielectric constant near the exciton resonance frequency can be described by^[3]^

$$\varepsilon\left( \omega\right)=\varepsilon_{\infty}\left( 1+\frac{\omega_{L}^{2}{-\omega}_{T}^{2}}{\omega_{T}^{2}-\omega^{2}-i\gamma_{X}\omega} \right) (1)$$

where $\varepsilon_{\infty}$ is the background permittivity; $\omega_{L}$ and $\omega_{T}$ are the longitudinal and transverse exciton frequencies, respectively; $\gamma_{X}$ is the decay rate of excitons. With the dielectric constant, the differential reflectance can be written by^[3]^

$$R\left( \omega\right)=A\left| \frac{\sqrt{\varepsilon\left( \omega\right)}-1}{\sqrt{\varepsilon\left( \omega\right)}+1} \right|^{2}+B (2)$$

where A and B are adjustable parameters for fitting the measured reflectance spectra. Here, the values of $\gamma_{X}$ were extracted from the linewidth of the exciton resonance through spectral fitting of the reflectance spectra. The fitted exciton resonance energies are shown in Figure 1b. Here, the values of $\gamma_{X}$ were extracted from the linewidth of the exciton resonance through spectral fitting of the reflectance spectra. The fitted exciton resonance energies are shown in Figure 1b.

To analyze the dispersion of exciton-polaritons, we used a coupled oscillator model for the coupled system of excitons and photons. Coupled oscillator Hamiltonian can be expressed as follows:^[4]^

$$\hat{H}\left( \theta\right)=\left( \begin{matrix} E_{X}+i\hbar\gamma_{X} & g \\ g & E_{ph}\left( \theta\right)+i\hbar\gamma_{ph} \end{matrix} \right) (3)$$

where $\gamma_{ph}$ is the decay rate of cavity photons; $E_{X}$ is the exciton energy; $E_{ph}(\theta)$ is the cavity photon energy; and $g$ is the coupling strength between the exciton and photon, which is half of Rabi splitting $(g=\hbar\Omega_{R}/2)$. From this Hamiltonian, we can derive the eigenvalues corresponding to the upper ($E_{+}$) and lower ($E_{-}$) polariton energies:^[4]^

$$E_{\pm}=\frac{E_{X}+E_{ph}}{2}+\frac{i\hbar\left( \gamma_{X}+\gamma_{ph} \right)}{2}\pm\frac{1}{2}\sqrt{\left( \hbar\Omega_{R} \right)^{2}+\left( E_{X}-E_{ph}+i\hbar\left( \gamma_{X}-\gamma_{ph} \right) \right)^{2}} (4)$$

The values of $\gamma_{ph}$ were extracted from the linewidth of pure photon modes near the polariton modes in the microcavity reflectance spectra.

**S3. Estimation of exciton oscillator strength**

To consider the spatial overlap between the active medium and the cavity photon mode, the Fabry-Pérot mode in the planar microcavity was calculated using a finite-difference time-domain method. From the calculated spectral reflectance, as shown in Figure S4b, the cavity resonance mode is observed as a narrow reflectance dip at the energy of 2.298 eV under normal incidence. At the energy of 2.298 eV, the electric field intensity distribution in the cross-sectional plane of microcavity is shown in Figure S4c. Figure S4d shows the spatial distribution of the electric field intensity and the refractive index within the cavity. While considering the planar microcavity geometry, we can calculate the spatial overlap ($\Gamma_{overlap}$) between the electric field and the excitons as the following equation:^[5]^

$$\Gamma_{overlap}=\frac{\int_{active} \varepsilon\left( r \right)\left| E\left( r \right) \right|^{2}dr^{3}}{\int_{cavity} \varepsilon\left( r \right)\left| E\left( r \right) \right|^{2}dr^{3}} (5)$$

where $\left| E\left( r \right) \right|^{2}$ is the intensity of the electric field and $\varepsilon\left( r \right)$ is the dielectric constant of materials. Taking into account the exciton oscillator strength and the spatial overlap, the vacuum Rabi splitting energy can be written by

$$\hbar\Omega_{R}\approx\sqrt{2\hbar\omega_{LT}\cdot\hbar\omega_{0}\cdot\Gamma_{overlap}} (6)$$

where$\hbar\omega_{LT} ({=\hbar\omega}_{L}-{\hbar\omega}_{T})$ is the longitudinal-transverse splitting of excitons, $\hbar\omega_{0}$is the exciton resonance energy. The longitudinal-transverse splitting of excitons is related to the exciton oscillator strength as follows: ^[5]^

$$\omega_{LT}=\frac{2\pi e^{2}}{\varepsilon_{\infty}\varepsilon_{0}m_{0}\omega_{0}}\left( \frac{f}{V} \right) (7)$$

where $f/V$ is the oscillator strength per unit volume; $\varepsilon_{\infty}$ and $\varepsilon_{0}$ is the background permittivity and the vacuum permittivity, respectively; $e$ is the electron charge; and $m_{0}$ is the free electron mass. Using the Equations (6) and (7), the exciton oscillator strength can be expressed as:

$$f/V\approx\left( \varepsilon_{\infty}\varepsilon_{0}m_{0}\Omega_{R}^{2} \right)/\left( 4\pi e^{2}\Gamma_{overlap} \right) (8)$$

**S4. Effect of electron-phonon coupling on exciton oscillator strength**

The effect of electron-phonon coupling has been considered to explain the decrease in the exciton oscillation strength with increasing temperature. Specifically, previous studies on InGaAs/GaAs multiple quantum wells have revealed a gradual decline in the exciton oscillator strength due to the increased phonon populations at elevated temperatures.^[6]^ In the case of MAPbI_3_ and MAPbBr_3_ perovskite materials, the Fröhlich interaction, indicating a coupling between electrons and longitudinal optical (LO) phonon, is dominantly attributed to the spectral broadening of the exciton resonances.^[7]^ Therefore, the temperature-dependent exciton oscillator strength $f(T)$ can be described by a modified Debye-Waller factor with the effective phonon mode as the following equation:^[5,8]^

$$f\left( T \right)\approx f_{0}\exp\left[ -\left\langle\boldsymbol{S} \right\rangle\coth\left( \frac{\left\langle\hbar\omega_{LO} \right\rangle}{2k_{B}T} \right) \right] (10)$$

where $f_{0}$ is a total exciton oscillator strength; $\left\langle\boldsymbol{S} \right\rangle$ is the Huang-Rhys factor; $\left\langle\hbar\omega_{LO} \right\rangle$ is the averaged LO phonon energy; and $k_{B}$ is the Boltzmann constant. $\left\langle\hbar\omega_{LO} \right\rangle$ is known to be ~20 meV for MAPbBr_3_.^[9]^ Using the modified Debye-Waller factor, we obtained the parameters of $\left\langle\boldsymbol{S} \right\rangle$and $f_{0}$ to be 0.18 and 11 × 10^24^, respectively, by fitting the exciton oscillator strengths experimentally obtained for the cubic phase, corresponding to the temperature range from 230 K to 297 K (blue dashed line in Figure 3b).

**S5. Effect of ferroelectric domains on exciton oscillator strength**

We employ the first-principles density functional theory calculation with the highly accurate full-potential linearized plane wave (LAPW) + local orbitals implemented in the ELK code. In the calculation, the Perdew-Wang local density approximation exchange correlational functional and 7 of maximum momentum of planewave basis $\times$ Muffin-tin radius are adopted.^[10]^ To study the exciton properties, including large polarization domains of the MAPbBr_3_, we suggest the simple model crystals for cubic semiconductors to provide the simplest and ideal semiconducting band structures (Figure S10). Within a first-principles approach, fully demonstrating the distinct phases of MAPbBr_3_ would require constructing a large system comprising at least ~960 atoms. To address this complexity, we adopted an approximation that captures the essential features of the system. It has been reported that in MAPbBr₃, the band edges, i.e., the conduction and valence band edges, are predominantly constructed by orbitals from two atoms, i.e., Pb and Br atoms.^[11,12]^ Considering the critical role of the band edges in excitonic properties, the full structure of MAPbBr₃ could be effectively reduced to a simplified minimal two-atom model crystal. Even though this calculation was performed with the simple two-dimensional lattice model, our model could successfully reproduce the effect of ferroelectricity on the exciton oscillator strength for the four types of electric polarization configurations. Indeed, the calculated exciton oscillator strength well reflects the experimental results. This agreement indicates that our approach effectively captures the essential physics related to the exciton oscillator strength in polar domains. Here, the five structural phases are defined as AF1 (anti-ferroelectric 1), AF2 (anti-ferroelectric 2), M (mixed), F (ferroelectric), and Cubic, which are categorized by the spatial arrangement of perturbative external electric fields, $E_{ext}\left( \boldsymbol{r} \right)=-\nabla\phi_{ext}\left( \boldsymbol{r} \right)$, simulating the polarization domain. It is notable that the $\phi_{ext}\left( \boldsymbol{r} \right)$ is supposed to be a solely significant variable to demonstrate the respective phases and their domains of MAPbBr_3_. The space-dependent potential, $\phi_{ext}\left( \boldsymbol{r} \right)$, is included in the Kohn-Sham effective Coulomb potential, $\phi_{KS;Coul}\left( \boldsymbol{r} \right)$, in the Kohn-Sham Hamiltonian, $(1/2\sum_{j} \left( -i\nabla_{j} \right)^{2}+\phi_{KS;Coul}\left( \boldsymbol{r} \right)+\phi_{KS;xc}\left( \boldsymbol{r} \right))$. Note that the $\phi_{KS;xc}\left( \boldsymbol{r} \right)$ is the exchange-correlational potential. The $\phi_{ext}\left( \boldsymbol{r} \right)$ is stably adopted consistently in both Muffin-tin and interstitial regions. The perturbative external electric fields are $E_{ext}\left( \boldsymbol{r} \right)=E_{0}\theta(\boldsymbol{r})\hat{\boldsymbol{y}}$, where the $\theta(\boldsymbol{r})$ denotes for the space-dependent sign function ($+1$ or $-1$) and the perturbative field strength, $E_{0}$, is fixed to be 0.002 atomic unit. The results of the calculation show a good agreement with the experiment, as shown in the main text. Since the polarization domain requires supercell structures, the phases AF1, AF2, M, and F are constructed with the $2\times2$, $1\times10$, $1\times10$, and $1\times10$ supercells, respectively. The first Brillouin zone is also sampled with $12\times12$, $14\times1$, $14\times1$, and $14\times1$ for respective phases. The dielectric functions are converged with respect to the unit cell calculation (cubic phase) with $24\times24$ sampled first Brillouin zone. In order to visualize the phases in the real space, we display the induced electric field $\boldsymbol{E}_{inc}\left( x,y,z=z_{c} \right)=-\nabla\left( \phi_{KS;Coul}\left( \boldsymbol{r} \right)\left. \right|_{\phi_{ext}}-\phi_{KS;Coul}\left( \boldsymbol{r} \right)\left. \right|_{\phi_{ext}=0} \right)_{\left\{ x,y,z=z_{c} \right\}}$. The $z_{c}$ is the vertical position of the model crystals. After the Kohn-Sham ground state calculation, we employ the Bethe Salpeter equation to estimate oscillator strengths and dielectric function of the excitons. The oscillator strength is defined as $f\sim\frac{1}{V}\left| \left\langle G\left| \hat{p} \right|X \right\rangle\right|^{2}/E_{X}$, where $\left| G \right\rangle$ and $\left| X \right\rangle$ denote for eigenvectors of the initial and exciton states, respectively; $\hat{p}$ is the momentum operator; and $E_{X}$ is the exciton eigenvalue.

**
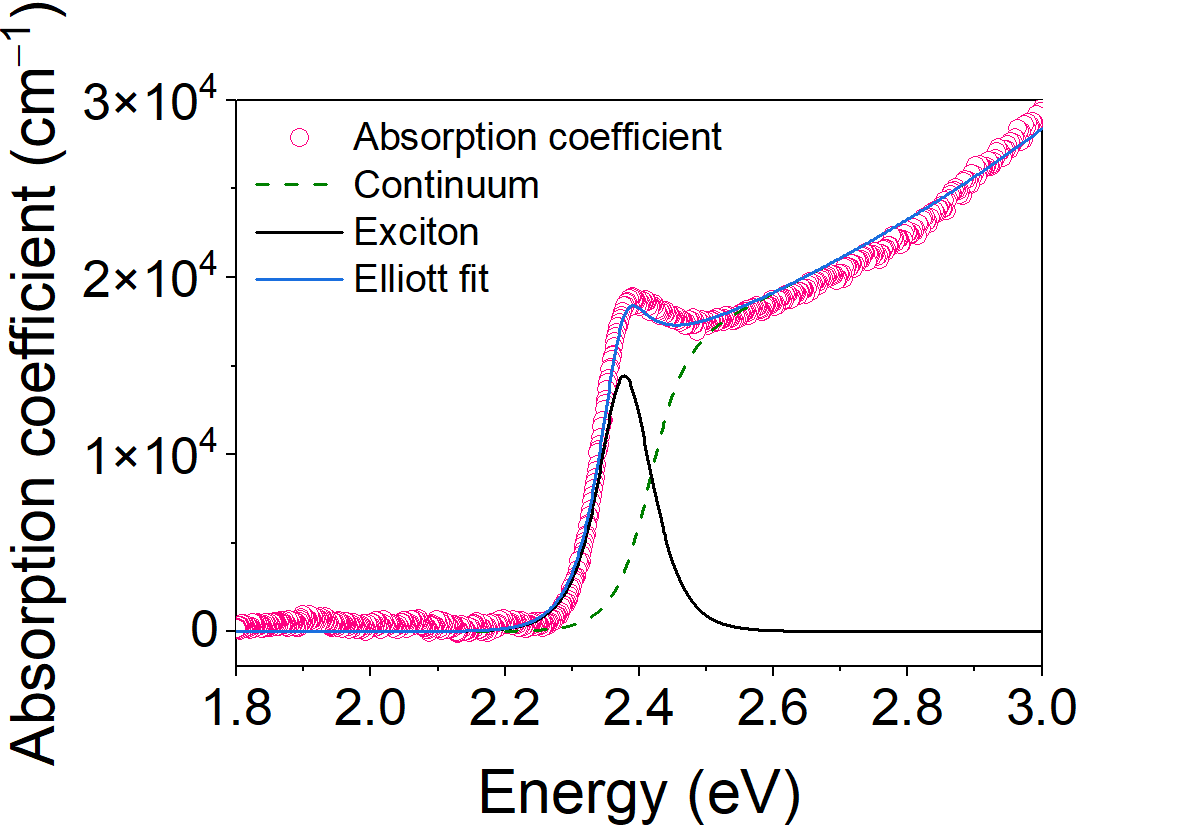
**

**Figure S1.** Absorption spectrum for MAPbBr_3_ films at 297 K. The experimental absorption spectrum (pink circles) is fitted using the Elliott model (blue solid line).^[13]^ Excitonic absorption (black solid line) and continuum band absorption (olive dashed line) are separately plotted.


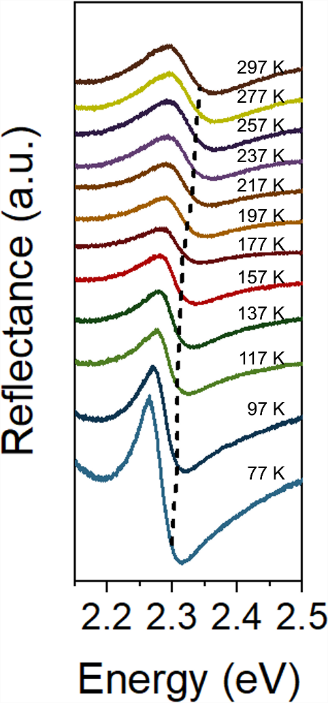


**Figure S2.** Temperature-dependent exciton energy of MAPbBr_3_ films. Temperature-dependent differential reflectance spectra plotted by a step of 20 K.


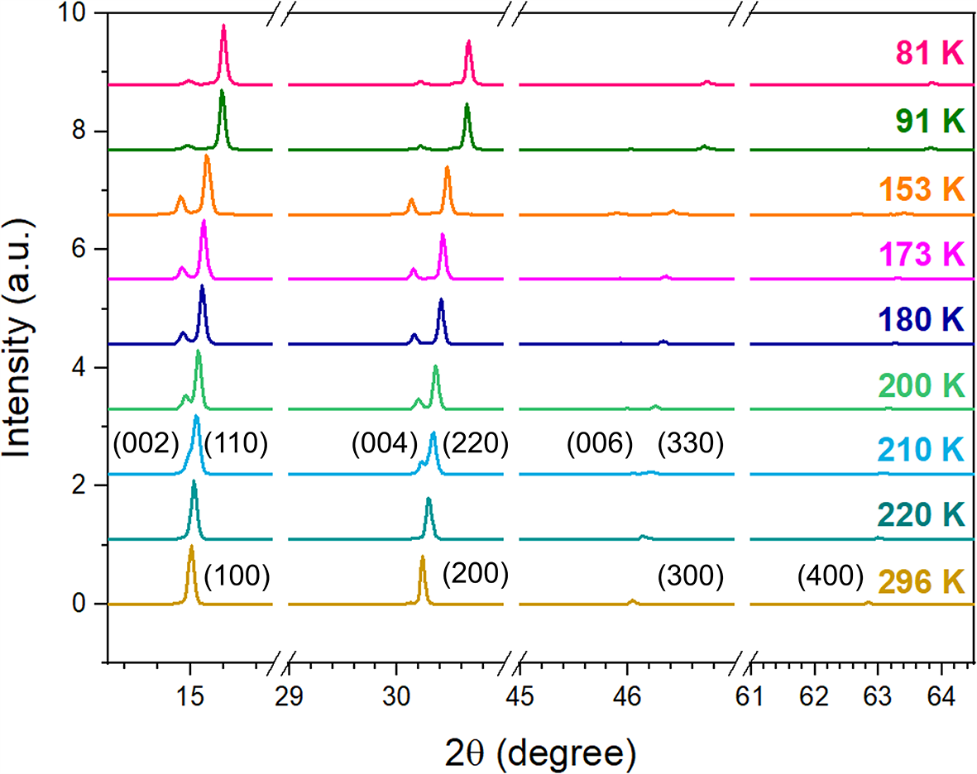


**Figure S3.** Phase-dependent X-ray diffraction (XRD) spectra of MAPbBr_3_ microplates. The XRD spectra measured at temperature ranges from 81 K to 296 K.


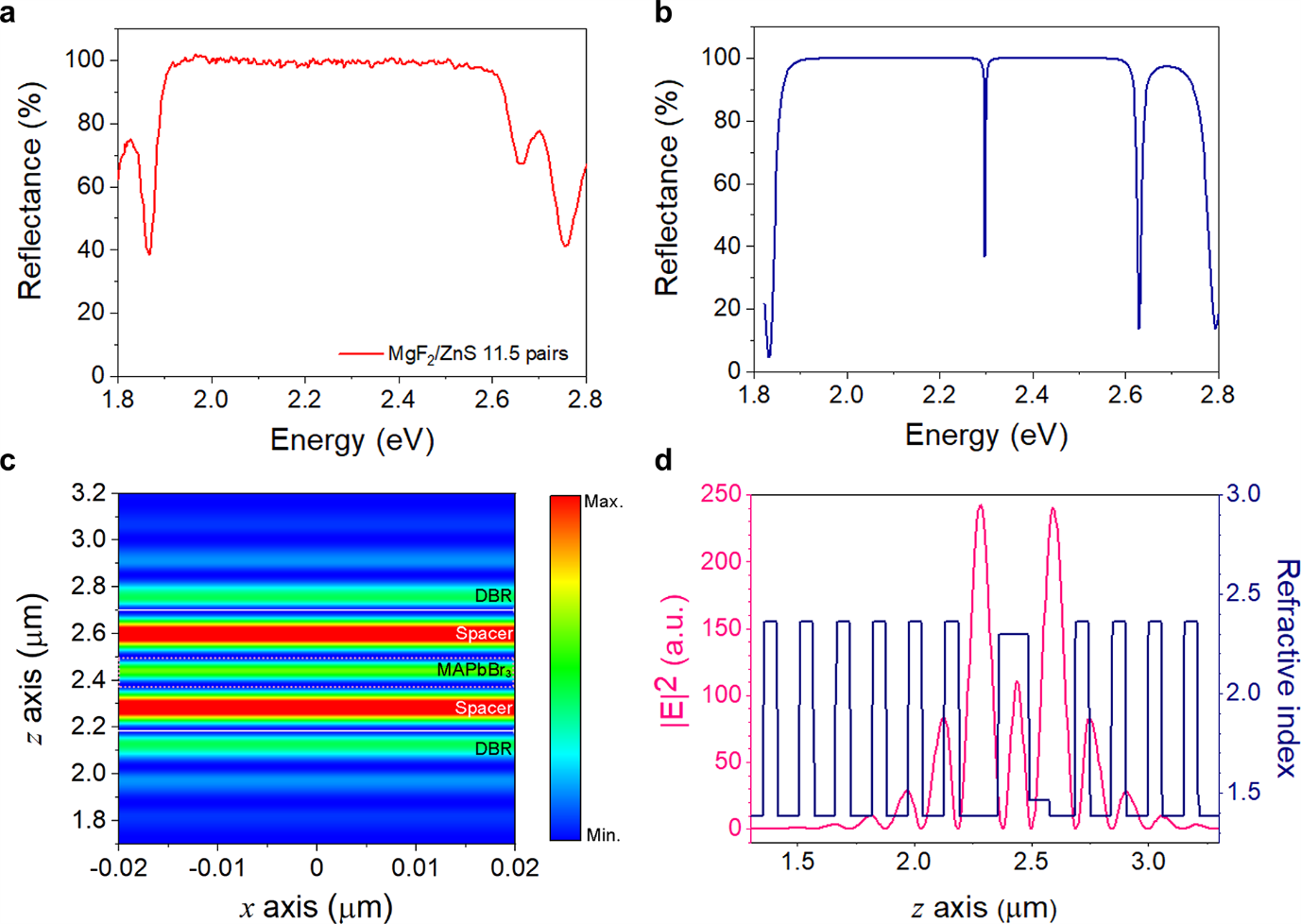


**Figure S4.** Design and simulation of MAPbBr_3_ optical microcavity. a) Reflectance spectrum of the fabricated bottom DBR consisting of 11.5 pairs of MgF_2_/ZnS using an e-beam evaporation system. b) Reflectance spectrum for the designed microcavity simulated by finite-difference time-domain method. The reflectance dip displays the sharp resonance at 2.298 eV with an FWHM of 3 meV. c) Electric field intensity profile for the designed microcavity. The MAPbBr_3_ film is embedded within the spacer layers of MgF_2_ and PMMA. d) Calculated electric field intensity distribution and refractive index profile along the *z*-axis.

**
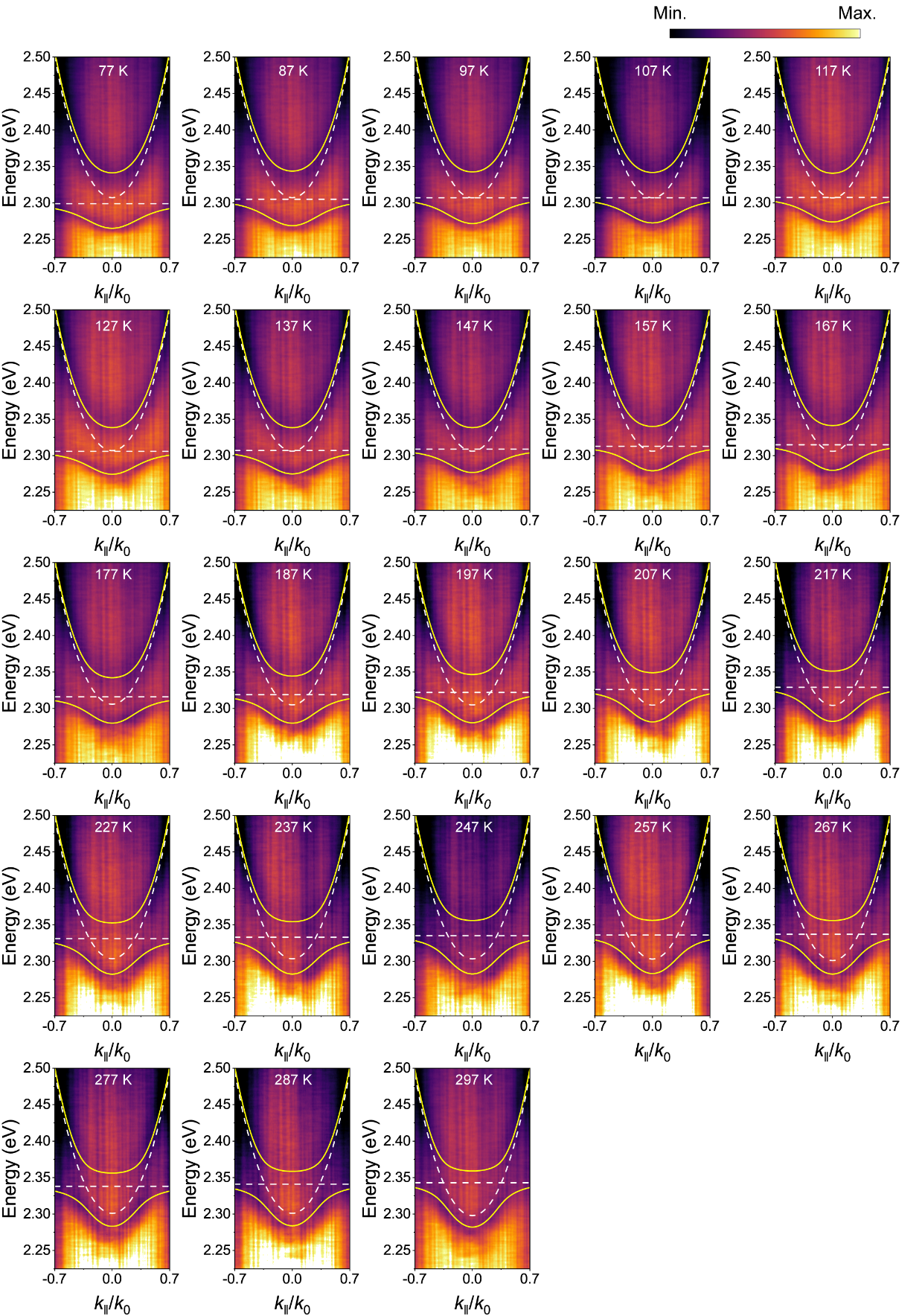
**

**Figure S5.** Temperature-dependent angle-resolved reflectance. Measurements were performed at the temperature range from 77 K to 297 K with a step of 10 K.


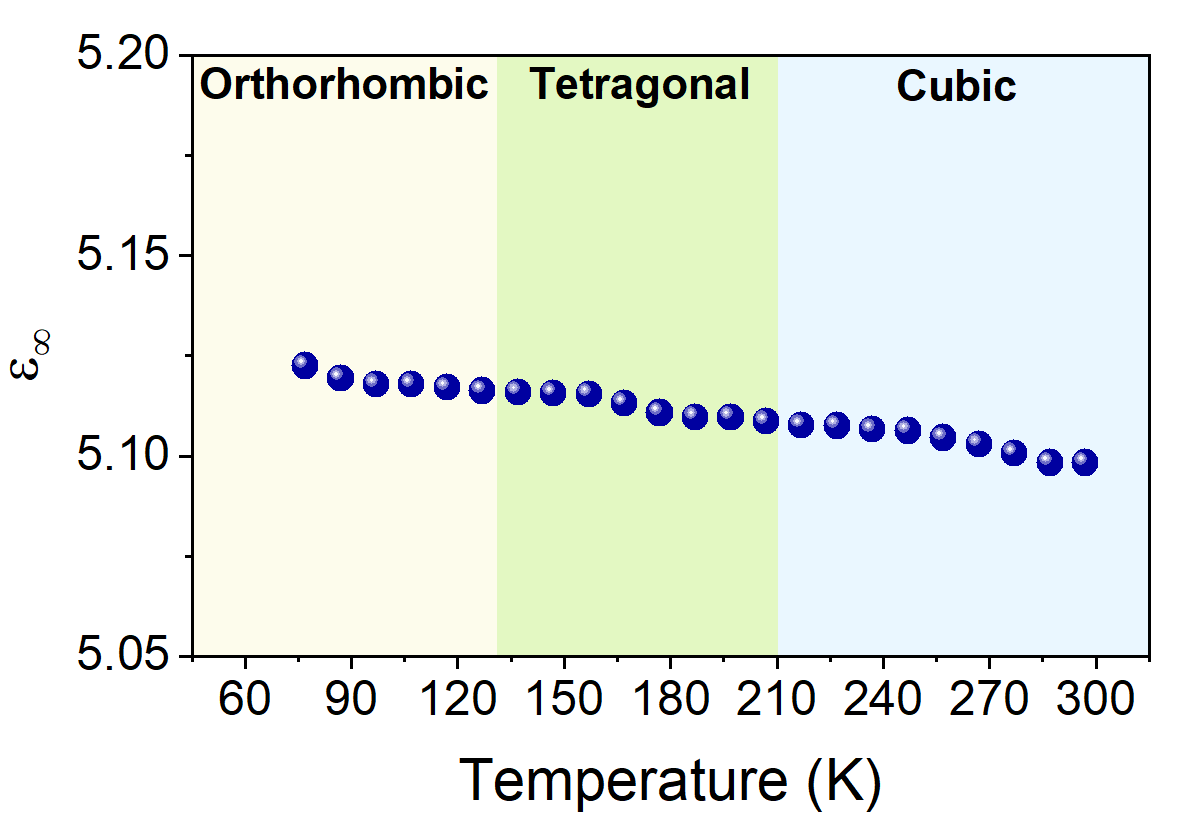


**Figure S6.** Temperature-dependent high-frequency permittivity. High-frequency permittivity obtained by fitting the cavity photon dispersion of the angle-resolved spectra at the temperature range from 77 K to 297 K.


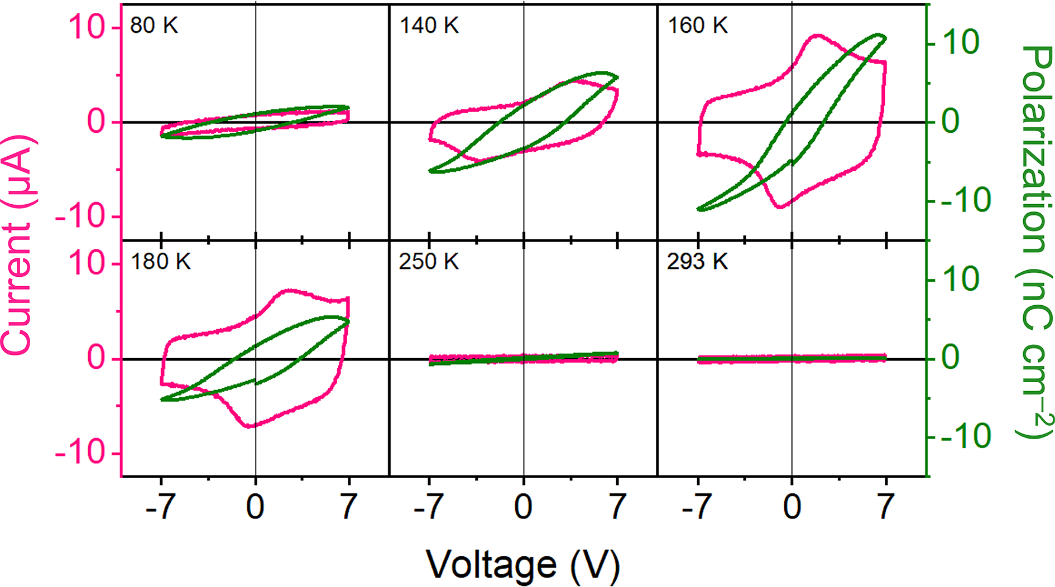


**Figure S7.** Temperature-dependent *I–V* and *P–V* hysteresis loops. Current versus voltage loops (pink curves) and the corresponding polarization loops (olive curves) at 80, 140, 160, 180, 250, and 293 K. Current peaks are observed at the temperatures of 140, 160, and 180 K, corresponding to the tetragonal phase.


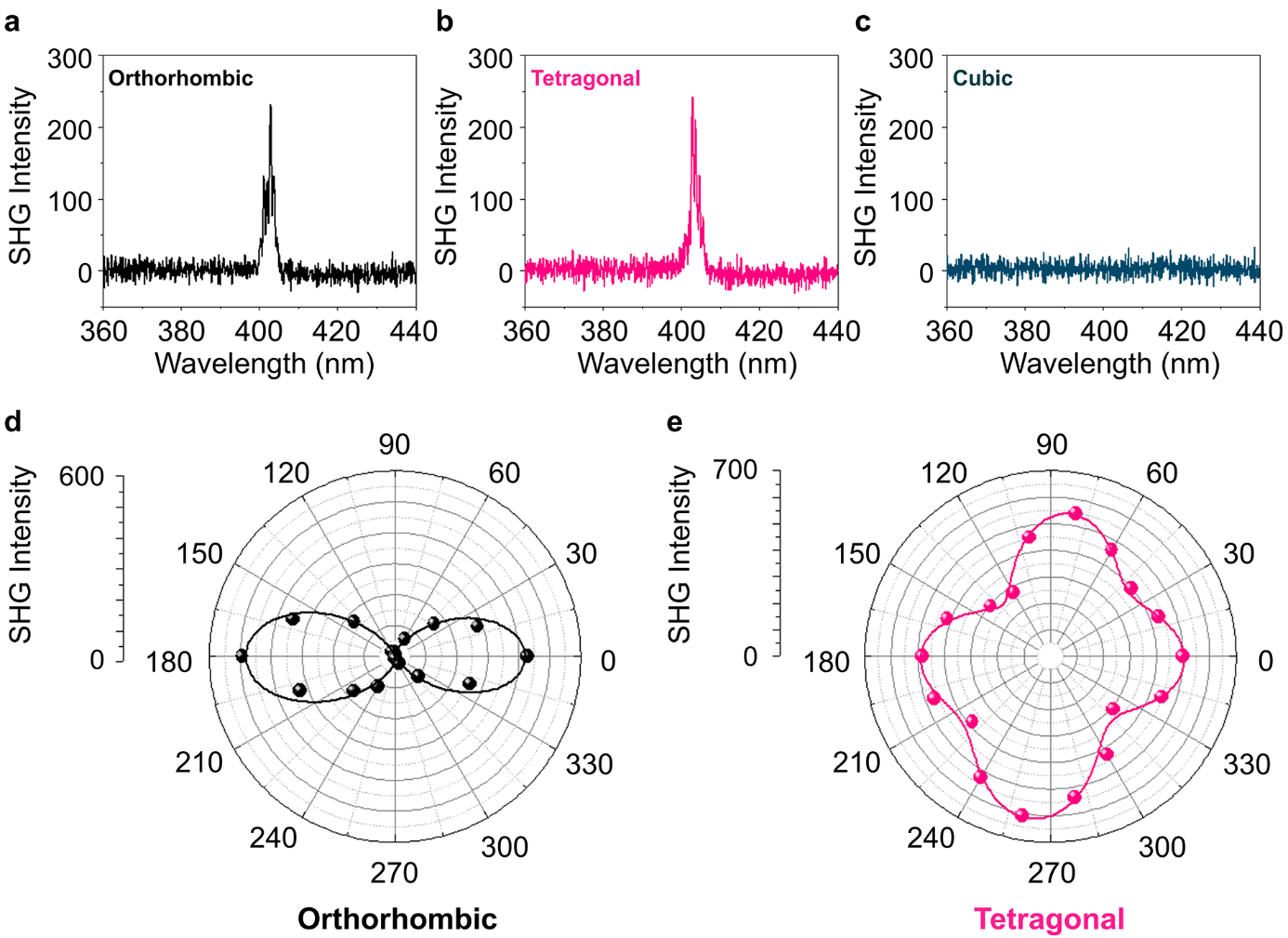


**Figure S8.** Phase-dependent second harmonic generation (SHG) in MAPbBr_3_. a–c) SHG spectra for orthorhombic (a), tetragonal (b), and cubic phases (c) under an excitation wavelength of 806 nm. d, e) Polar plots of the SHG intensity for orthorhombic (d) and tetragonal phases (e) as a function of the polarization angle of the excitation.


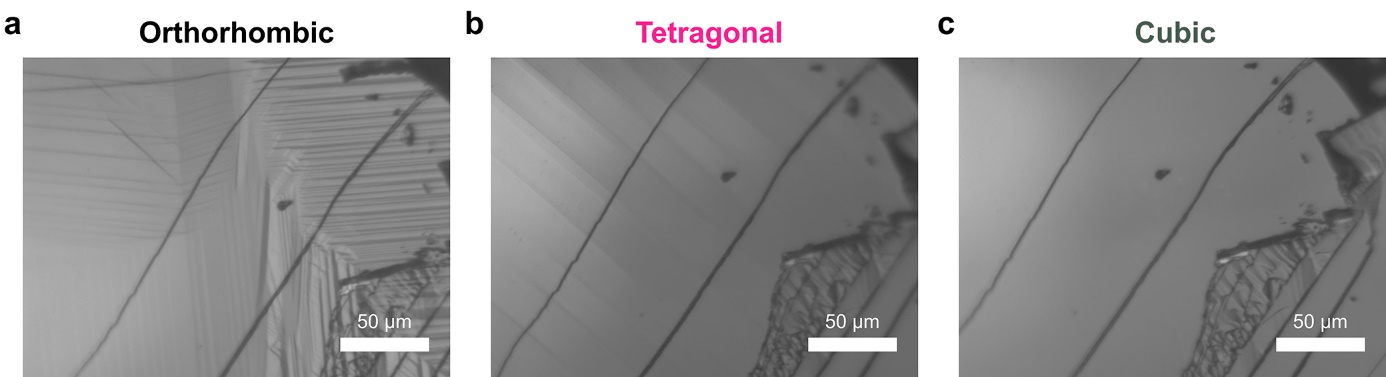


**Figure S9.** Phase-dependent surface morphologies of MAPbBr_3_ crystals. a–c) Optical microscopic CCD images of cleaved surfaces for orthorhombic (a), tetragonal (b), and cubic phases (c).


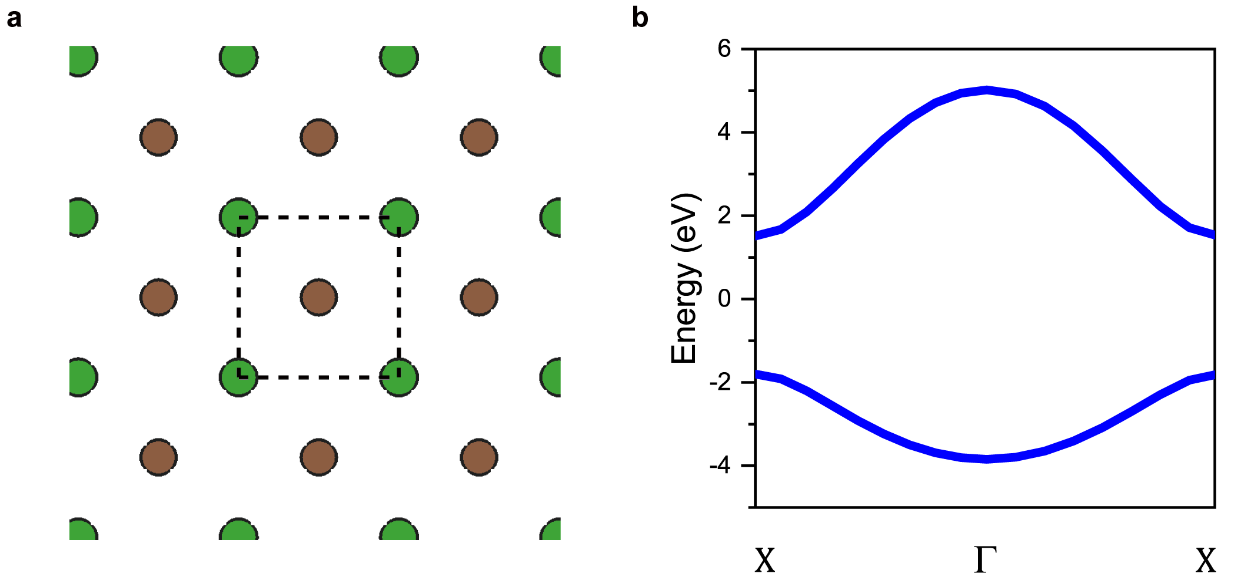


**Figure S10.** Schematic simple model crystal and calculated band structure. a) The simple model crystal with Gedanken atoms whose artificial atomic numbers, 0.8 (green) and 1.4 (brown) with tightly bounded s-wave electrons, are given to reproduce proper semiconducting band structures. The brown and green atoms represent Br and Pb atoms, respectively. The dashed box indicates a unit cell of the model crystals with 5.67 atomic unit. b) The simplest and ideal semiconducting band structure of the model crystal.


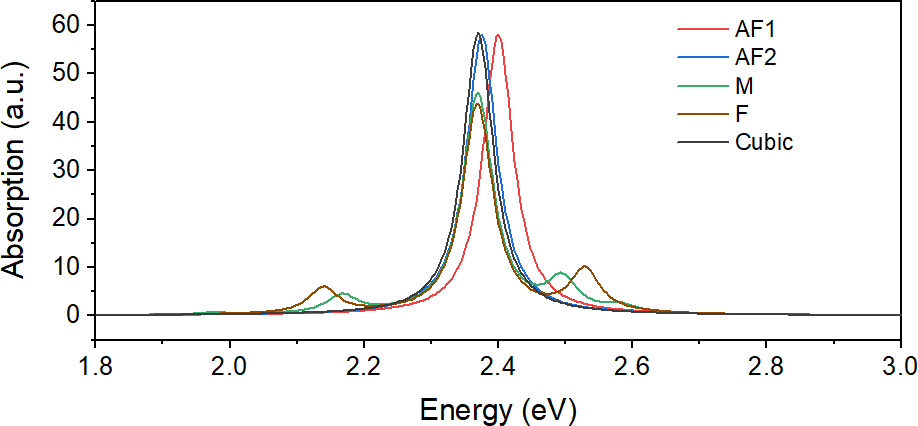


**Figure S11.** Calculated absorption spectra from the imaginary dielectric function of exciton states. The dielectric function is converted from the optical conductivity.

**Reference**

[1] A. Poglitsch, D. Weber, *J. Chem. Phys.* **1987**, *87*, 6373.

[2] C. Chen, X. Hu, W. Lu, S. Chang, L. Shi, L. Li, H. Zhong, J.-B. Han, *J. Phys. D: Appl. Phys.* **2018**, *51*, 045105.

[3] J.-W. Kang, B. Song, W. Liu, S.-J. Park, R. Agarwal, C.-H. Cho, *Sci. Adv.* **2019**, *5*, eaau9338.

[4] S. Haroche, D. Kleppner, *Phys. Today* **1989**, *42*, 24.

[5] S. H. Gong, S. M. Ko, M. H. Jang, Y. H. Cho, *Nano Lett.* **2015**, *15*, 4517.

[6] B. Zhang, S. S. Kano, Y. Shiraki, R. Ito, *Phys. Rev. B* **1994**, *50*, 7499.

[7] A. D. Wright, C. Verdi, R. L. Milot, G. E. Eperon, M. A. Pérez-Osorio, H. J. Snaith, F. Giustino, M. B. Johnston, L. M. Herz, *Nat. Commun.* **2016**, *7*, 11755.

[8] S. Nomura, T. Kobayashi, *Phys. Rev. B* **1992**, *45*, 1305.

[9] M. Nagal, T. Tomioka, M. Ashida, M. Hoyano, R. Akashi, Y. Yamada, T. Aharen, Y. Kanemitsu, *Phys. Rev. Lett.* **2018**, *121*, 145506.

[10] J. P. Perdew, Y. Wang, *Phys. Rev. B* **1992**, *45*, 13244.

[11] W. Geng, L. Zhang, Y.-N. Zhang, W.-M. Lau, L.-M. Liu, *J. Phys. Chem. C* **2014**, *118*, 19565.

[12] J. Su, D. Luise, I. Ciofini, F Labat, *J. Phys. Chem. C* **2023**, *127*, 5968.

[13] M. Saba, M. Cadelano, D. Marongiu, F. Chen, V. Sarritzu, N. Sestu, C. Figus, M. Aresti, R. Piras, A. G. Lehmann, C. Cannas, A. Musinu, F. Quochi, A. Mura & G. Bongiovanni, *Nat. Commun.* **2014***, 5*, 5049.
